# Supplementary material for: New perspectives on university quality assessment: A Mamdani Fuzzy Inference System approach
Source: PLoS One. 2025 May 19;20(5):e0321013. doi: 10.1371/journal.pone.0321013 (PMC12088516; doi:10.1371/journal.pone.0321013)
Supplement: Attachment — Submitted filename: ReviewforPlosONE.pdf [file pone.0321013.s002.pdf]

Review

This paper discusses the quality of higher education in general with the specific case of the city-state of Madrid, Spain. Even so, the case seems to represent all European countries based on the considerations submitted to quality assurance institutions, of course with the rules attached to each other.

1. However, it is undeniable that each country or university has its own competence, and perhaps determining the measurement variables must consider the general and specific characteristics for it, which are not explained from the perspective of the interests of each university, then a methodology is proposed to assess the quality of the university.
2. Perhaps, not all causal patterns in language are appropriate to the variables considered, if not directly related to the pattern is the decision that causes the value of the results.
3. The results may not be directly quantitative, but the quantity is a reflection of a methodology that the measurement in the study has been successful. Perhaps, the author considers expanding the proposed methodology, by reinterpreting the measurement variables.
4. Although this study is a view of what is being studied, the synchronization of the existing problems between the potential objectives so that the conclusions answer the problems raised by the author.

Overall this paper is good. Thank you for the opportunity to read it.
